# Oscillation of the velvet worm slime jet by passive hydrodynamic instability

**DOI:** 10.1038/ncomms7292

**Published:** 2015-03-17

**Authors:** Andrés Concha, Paula Mellado, Bernal Morera-Brenes, Cristiano Sampaio Costa, L Mahadevan, Julián Monge-Nájera

**Affiliations:** 1 School of Engineering and Sciences, Adolfo Ibañez University, Diagonal las Torres 2640, Peñalolen, Santiago 7941169, Chile; 2 Laboratorio de Genética Evolutiva, Escuela de Ciencias Biológicas, Universidad Nacional de Costa Rica, 86-3000 Heredia, Costa Rica; 3 Department of Zoology, Institute of Bioscience, Universidade de Sao Paulo, Sao Paulo, Sao Paulo 11461, Brazil; 4 School of Engineering and Applied Sciences, Harvard University, Cambridge, Massachusetts 02138, USA; 5 Department of Physics, Harvard University, Cambridge, Massachusetts 02138, USA; 6 Tropical Biology, Universidad de Costa Rica, 2060 San José, Costa Rica

## Abstract

The rapid squirt of a proteinaceous slime jet endows velvet worms (Onychophora) with a unique mechanism for defence from predators and for capturing prey by entangling them in a disordered web that immobilizes their target. However, to date, neither qualitative nor quantitative descriptions have been provided for this unique adaptation. Here we investigate the fast oscillatory motion of the oral papillae and the exiting liquid jet that oscillates with frequencies *f*~30–60 Hz. Using anatomical images, high-speed videography, theoretical analysis and a physical simulacrum, we show that this fast oscillatory motion is the result of an elastohydrodynamic instability driven by the interplay between the elasticity of oral papillae and the fast unsteady flow during squirting. Our results demonstrate how passive strategies can be cleverly harnessed by organisms, while suggesting future oscillating microfluidic devices, as well as novel ways for micro and nanofibre production using bioinspired strategies.

## Abstract (web summary)

The velvet worm emits a rapidly oscillating jet of proteinaceous slime to capture prey. Here, Concha *et al*. combine high-speed videography and a physical simulacrum to establish that this passive mechanism is the result of elastohydrodynamic instability during high-speed flow through the oral papillae.

## Graphical abstract

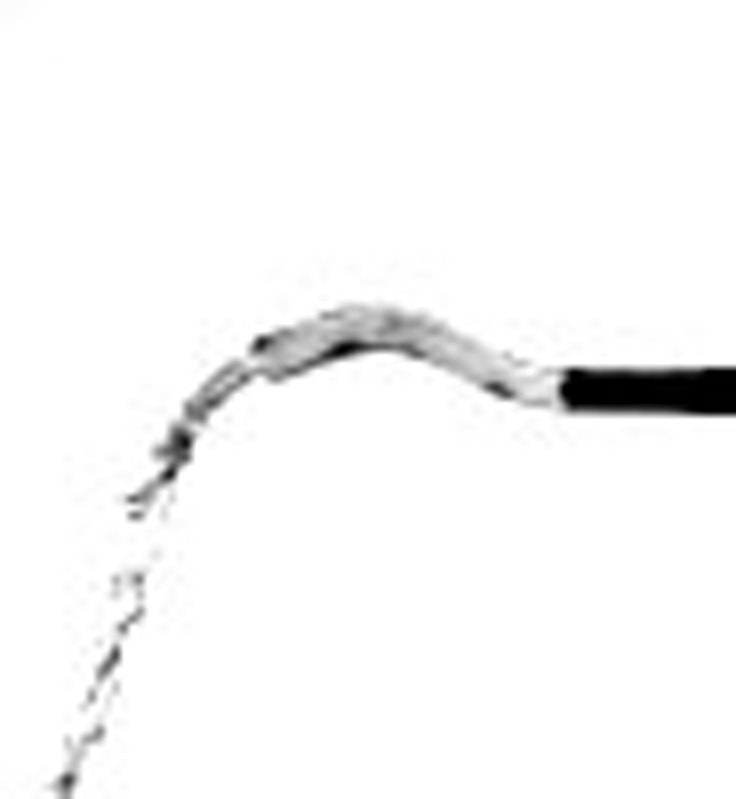

Rapid motions in nature are seen in a variety of situations associated with escape and predation. Extreme examples include the chameleon tongue that uses an unusual muscle-spring configuration to capture prey^1^, and the venus flytrap that stores elastic energy and uses an instability for the rapid closure of its leaf^2^. The velvet worm is an unusual example of how an organism projects itself by squirting a jet of slime in an oscillatory fashion, not only for capturing prey^3^, but also for defence^4^,^5^,^6^,^7^,^8^. Animals use squirting mechanisms for elimination of waste products (for example, urination in vertebrates); reproduction (gamete expulsion); communication (for example, pheromone excretion in mammals); locomotion (for example, squids); defence (for example, horned lizards); and hunting (for example, archer fish)^9^,^10^,^11^,^12^,^13^. These jets are normally directed in a straight line. We are aware of only three exceptions: velvet worms (Phylum Onychophora), spitting spiders (*Scytodes* spp.) and spitting cobras (*Naja* spp.). Spitting spiders oscillate their fangs^14^, and spitting cobras actively oscillate their heads^15^. Despite having been a subject of study for over a century, the mechanism underlying the rapid oscillatory squirting of slime by the velvet worm remains a mystery^4^,^16^,^17^. Indeed, Darwin even hypothesized the creation of the disordered web as a potential origin for the evolution of spider webs^18^.

In this article, we demonstrate that the fast oscillations of the jet slime and papillae in Onychophora are the result of a syringe-like system that, by means of a geometric amplifier, allows for fast squirt using slow muscular contraction. This fast flow activates an elastohydrodynamical instability that explains the fast papilla oscillations during defence and attack. We present a physical simulacrum that reproduces the instability in the same range of parameters of the natural system opening new venues for self-supported microfluidic devices and applications^19^,^20^,^21^,^22^.

## Results and Discussion

### Worm attack kinematics

**Figure 1:**
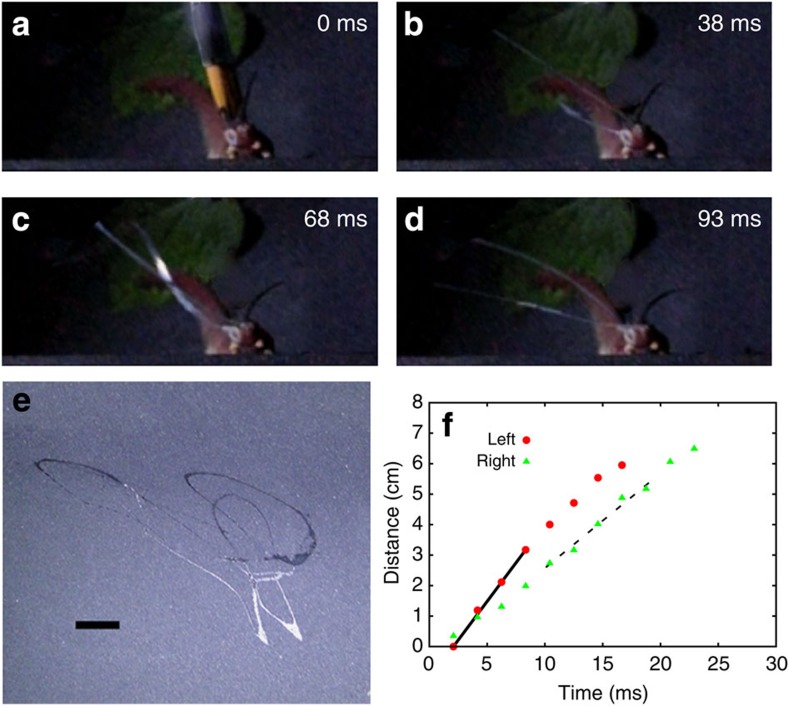
Slime jet oscillations and jet speed during worm attack. Giant red velvet worm *Peripatus solorzanoi* used to record the squirting process. Full-body length is ~17.5 cm. In **a**, a soft paintbrush used to activate its attack is shown, and it was digitally removed in the other snapshots for clarity. (**a**–**d**) Different stages of the attack recorded at 480 f.p.s. The active part of the attack is completed in Δ*t*_squirt_~65 ms. (**e**) Slime pattern generated on a wall of the foam tunnel used to keep worms at focal distance (scale bar, 1 cm). Three or more oscillations of the slime jet are the typical outcome. (**f**) Liquid jet tip position as a function of time for the squirt. For this data, we used two cameras: one at 30 f.p.s. and the other at 480 f.p.s. The two cameras configuration allowed us to compute the jet velocity. The solid line corresponds to a squirt speed of *V*=5.0 m s^−1^ and the dotted line to a squirt speed of *V*=3.2 m s^−1^. Solid red dots and green triangles correspond to left and right papilla, respectively.

To capture the dynamics of the squirting process, we filmed several worm attacks (Supplementary Movies 1,2,3). In Fig. 1a–d, we show a series of snapshots of an attack recorded using high-speed imaging (480 frames per second a.k.a. f.p.s.), with the average duration of a squirt for all specimens being Δ*t*_ave_=0.064±0.005 s (Supplementary Table I; Supplementary Note 2). By tracking the motion of the tip of the jet shown in Fig. 1 and Supplementary Fig. 2, as a function of time, we found that the typical jet speed *V*~3–5 m s^−1^. Furthermore, we see that the squirt does not remain oriented but instead sprays an entire region as shown in Fig. 1e. These measurements raise the natural question of the spatio-temporal evolution of the liquid jet and its control by the worm.

### Anatomy of the ejecting system

**Figure 2:**
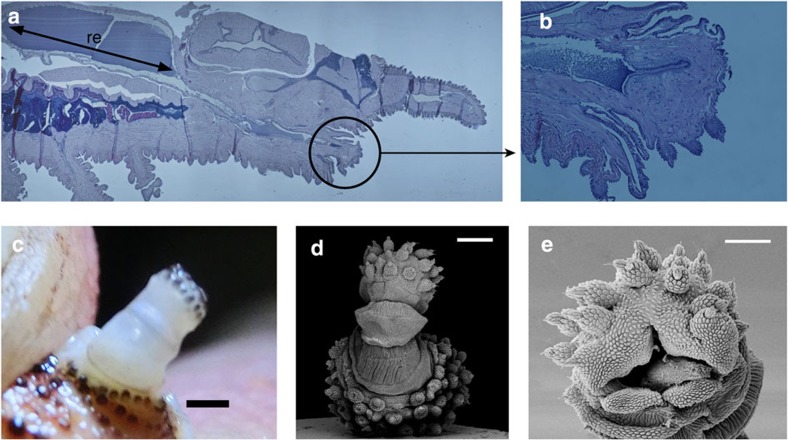
Squirt system and papilla structure. (**a**) A haematoxylin and eosin-stained section of a *Peripatus solorzanoi*. The squirt system is composed of a large slime reservoir, re, whose length is depicted by a black arrow. The reservoir has a large diameter compared with the diameter of the duct that connects it with the oral papilla (black circle). (**b**) Longitudinal cut of an oral papilla of a *Peripatus solorzanoi*. Its wrinkled surface and sphincter-like tissues surrounding the inner duct are shown (scale 4mm). The black substance inside the duct corresponds to remnants of the slime. (**c**) Structure of a fresh oral papilla (red *Peripatus solorzanoi*) when deployed. Its semi-transparent and wavy structure is apparent (scale bar, 1 mm). (**d**) Oral papilla accordion structure of a *Epiperipatus acacioi* (scale bar, 200 μm). In this case the hinged accordion structure is resolved by scanning electron microscopy (Leica Leo 440) taken at 15 kV after coating with gold (~20 nm) in a Denton Desk IV gold sputter model. This specimen was used to confirm that the accordion-like structure present in *Peripatus solorzanoi* is generic to Onychophora regardless of its size. (**e**) Opening of the oral papilla *D*~350 *μ*m and *d*~200 *μ*m (scale bar, 100 *μ*m).

Actively controlled muscular action has long been invoked as the natural explanation for the spectacular way in which these worms quickly squirt slime and continues to be the favoured mechanism^16^. However, papillar oscillations are fast (Fig. 1; Supplementary Table 1 and Supplementary Note 2) in comparison with any other motion of the worm (*f*_papilla_/*f*_walking_~30–60) and with known time scales (~0.5 s) for the fastest muscles in the worm^23^, suggesting a conceptual difficulty with this hypothesis. Therefore, we examined the anatomy of the whole-squirt system and surrounding tissues (Fig. 2; Supplementary Fig. 1). During squirting, the oral papilla extends from its folded shape to its full length (Fig. 2) of up to *L*~6 mm (Supplementary Fig. 2). In Fig. 2a, we see a large reservoir region (re) where slime is stored and a narrow duct that ends at the oral papilla, a syringe-like geometry that facilitates the acceleration of the slime for the fast squirt. In Fig. 2a,b, we show that muscle fibres in the oral papilla are similar to those found in the legs, but fewer in number. Figure 2a,b and Supplementary Fig. 1 also show that muscular fibres found in papilla tissues are consistent with their directional function, with some being annular, typical of sphincter-like systems. The relaxed papilla has an accordion shape (Fig. 2c,d) that is unfolded just before the squirting process, and can thus be easily packed while also having an inhomogeneous bending rigidity, with soft spots that make papillae more pliable and susceptible to bending as slime is squirted.

Our anatomical findings are consistent with earlier evidence that slime papilla are modified limbs^24^,^25^ with a nervous system similar to that in their legs^26^. Detailed descriptions of the Onychophora muscular system^23^ show that the fastest muscles are located in the jaw with typical twitch time scales ~0.5 s, which, while fast for this primitive worm, are nearly 25 times slower relative to the papillary oscillation time scale ~20 ms. Given that the legs and the papillary muscles are even slower, consistent with the primitive nature of these worms^5^,^27^,^28^, we are left with an obvious question—how are rapid changes in direction that occur over a time scale of a few milliseconds possible without the existence of any specialized rapid muscular actuation or neural control?

### Physical mechanism

A way around this conundrum is to realize that a physical mechanism can drive the rapid and nearly chaotic oscillations of the papilla just as a garden hose pipe oscillates when water squirts out of it rapidly. Indeed, the inertial effects associated with the exiting fluid jet drive the elastic hose pipe to flutter, a subject that has been well studied experimentally and theoretically at the macroscale^29^. In the current microscopic setting, the interplay between fluid forces and the papilla elasticity produce the characteristic oscillatory waving motion used to capture prey, and obviate the need for any fast moving controlled muscles. However, this mechanism requires fluid inertia to play a critical destabilizing role, that is, the ratio of inertial to viscous forces characterized by the Reynolds number Re=*VR*/*ν*>1 (where *V* is the characteristic fluid speed, *ν* the fluid kinematic viscosity and *R* the tube radius). In microfluidic geometries where typical sizes are small, unless the velocities are sufficiently large, inertial effects are unimportant.

Our microscopy studies (see Fig. 2a) show that the squirting system has a reservoir that contracts slowly driving the slime through a small duct that runs close to the centre of the oral papilla (Supplementary Figs 1 and 3). This geometric amplifier can lead to an increase in the speed during squirting. This is functionally similar to structures found in the chelicerae of spitting spiders^14^, and suggests that this geometric amplifier mechanism could be relevant for a variety of other squirting organisms. Our observations are in contrast with previously reported studies^30^ that have persisted into the modern literature^31^, where no mention of the cross-section reduction has been made. Our micrographs show muscular structures (Fig. 2; Supplementary Fig. 1) around the slime reservoirs, which are functionally consistent with the contraction of this organ. These structures resemble the design of radial tires where a fibre network is used to reinforce the wall^32^, consistent with detailed study of muscular fibres at reservoir level^33^. Measurements of the squirted volume (Supplementary Notes 1 and 3; Supplementary Fig. 4) and reservoir geometry show that the contraction ratio *δR*_re_/*R*_re_<0.03 (with *R*_re_~2 mm) changes in about 0.1 s, enough to produce speeds of *V*~5 m s^−1^, so that the Reynolds number Re~2,700 (Supplementary Note 6). Since we do not see perfect synchronization between liquid jets coming from different papilla (Fig. 1a–d; Supplementary Table I; Supplementary Movies 1 and 2), whole-body contraction as the main driving force in squirting^33^ is unlikely.

This leads to the conclusion that the instability arises due to a competition between fluid inertia and elastic resistance. When a liquid moves steadily through a flexible pipe at small *V*, flow-induced damping prevents any oscillations from growing. For large enough *V*, centrifugal and Coriolis forces make the pipe unstable for fluid speeds *V*>*V*_c_. In the limit when the effects of gravity can be neglected (Supplementary Notes 5–7; Supplementary Fig. 8), a simple scaling argument allows us to estimate the frequency of oscillations *f* by balancing the stabilizing elastic bending resistance with the destabilizing inertial forces, that is,


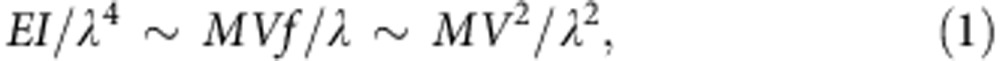


where *EI* is the bending stiffness, *λ*~2*L* is the approximate oscillation wavelength for the cantilevered papilla and *M* is the mass density per unit length of the fluid in the pipe. This yields


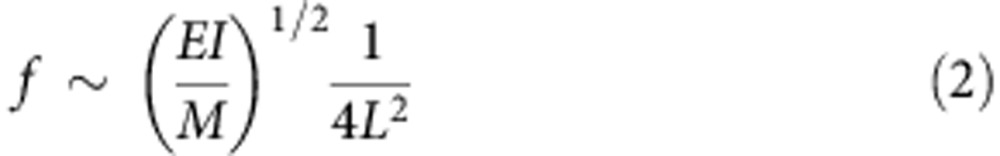


Similarly, a typical speed scale can be estimated as *u*_0_~*fλ*=(*EI*/*M*)^1/2^(1/*L*). For the specimen shown in Supplementary Fig. 2, the oscillation frequency *f*~58 Hz, papilla length *L*=6.0 mm, papilla outer diameter *D*=1.0 mm and papilla inner diameter *d*=0.5 mm, from where *β*~0.25. The measured frequency allows to estimate the effective Young’s modulus of the papilla to be *E*~20 kPa consistent with measurements using small magnets to pull the papilla that yields *E*~40 kPa (Supplementary Table 2), and thence the typical speed *u*_0_~0.5 m s^−1^. The precise critical speed *V*_c_ depends on boundary conditions, which for the cantilever case gives *V*_c_~2*πu*_0_=3 m s^−1^. At this critical speed, stability is lost via a Hopf type bifurcation^29^ (see Supplementary Note 5, 6 for further information).

For unsteady flows, such as when the jet is being accelerated inside the flexible papilla, the fluid acceleration d*v*/d*t* can destabilize the system at even lower jet speeds (see Eqs. 3, 4 in Methods and Supplementary Fig. 8). Our measurements show that *V*~3.2–5.0 m s^−1^. Therefore, even without muscular action the papilla will become unstable due to a simple physical instability^29^.

### Synthetic simulacrum

**Figure 3:**
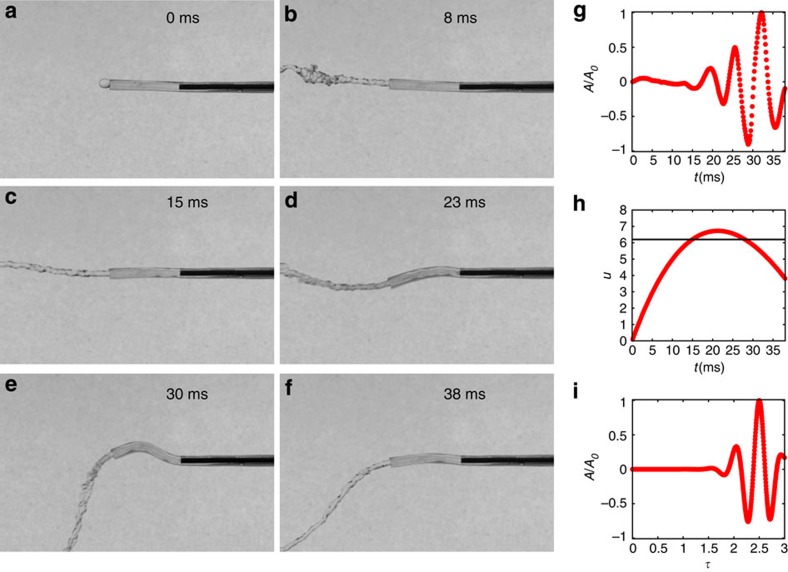
Bioinspired physical simulacrum of the oral papilla. (**a**–**f**) A soft elastic cantilevered tube made out of polydimethylsiloxane (PDMS) becomes unstable as the fluid flowing through it increases its speed. Its height is *h*=1.42 mm, width *w*=1.60 mm, length *L*=9.5 mm and hole diameter is 0.81 mm. The fluid used in this experiment was water. (**g**) The vertical motion of a point at the centre line of the hose close to the tip. As fluid speed is increased, oscillations develop and grow. The vertical axis is the dimensionless ratio between oscillation amplitude *A* and its maximum *A*_0_. (**h**) During an emptying cycle, the fluid speed varies as a function of time, with the dimensionless speed *u*ε[0.0,6.9], with *u*=*V*/*u*_0_. Horizontal black line depicts the theoretical threshold for instability in the case of steady flow. (**i**) Numerical simulations of the squirting dynamics associated with the dynamics of emptying shown in **h** using the governing equations. As in **g**, the vertical axis is the ratio between oscillation amplitude *A* and its maximum *A*_0_. The horizontal axis is the dimensionless time *τ*=*t*/*τ*_0_, where *τ*_0_ is the bending time scale.

To show that it is indeed possible to drive these oscillatory instabilities on small scales, we made a synthetic papilla out of a soft elastomer (see Methods) in the form of a flexible micropipe with a rectangular cross-section that defines an oscillation axis for its softest bending mode. Our pipe was moulded out of polydimethylsiloxane (PDMS) with a Young’s modulus *E*=288 kPa (Supplementary Figs 5–7; Supplementary Note 4), with thickness *h*=1.42 mm, width *w*=1.60 mm, inner diameter *d*=0.81 mm and length *L*=9.5 mm. Our model system is simpler than the natural one in at least two aspects: there is no roughness along the inner part of the duct or an external accordion-like geometry. Our experimental results (Fig. 3; Supplementary Fig. 5; Supplementary Note 5; Supplementary Movie 4) show that synthetic papilla becomes unstable and oscillates when the liquid reaches a speed of *V*_c_=8.6 m s^−1^. This occurs in the same range of dimensionless parameters that for the natural organ in agreement with theoretical predictions^29^, and shows that fast muscular action at the papillary level is unnecessary for oscillations. From measurements of external diameters (Fig. 2d), we found that *B*=*EI* can locally change up to 1/10 of the stiffness corresponding to a uniform soft papilla of diameter *D*_0_, showing that the accordion-like microstructure will lower *V*_c_. Microtubes with accordion-like shapes made out of the same polymer were made varying the degree of external roughness. Our data shows that *V*_c_ lowers as we increase the amplitude of external roughness (Supplementary Note 6; Supplementary Fig. 9). The flow of liquids in pipes with inner roughness has been extensively studied^34^,^35^. The effect of inner pipe imperfections is to decrease the effective cross-section of the tube, as well as to anticipate the transition to turbulence^35^. All these effects together lower *V*_c_, being the most important for the fluid regime described, the accordion-like modulations of the tube. Naturally, this experiment also suggests a prototype of a fluid driven micromechanical actuator^36^,^37^,^38^.

Our observations, minimal theory and physical mimic show how velvet worms can spray a web rapidly to entangle their prey without resorting to a complex neuromuscular control strategy. Instead, they can simply harness the instability associated with rapid flow through a long soft nozzle that causes it and the exiting jet to oscillate. As this jet solidifies rapidly, it entraps the object dynamically. While there is a superficial similarity to spider webs, we see that here dynamics is of essence. Our findings and synthetic model might also pave the way to self-supported flexible microfluidic devices that can take advantage of similar instabilities to produce a variety of products such as microdrops^22^,^39^,^40^,^41^, and non-woven fibre structures^19^.

## Methods

### Sample collection

Specimens of *Peripatus solorzanoi* (*n*=3) were collected in Guayacan, Siquirres, Costa Rica (10°3′21.38′′ N, 83°32′44.04′′ W, 500 m.a.s.l.). The specimen of *Epiperipatus acacioi* (*n*=1) was collected in Minas Gerais, Brazil (20°22′44′′ S, 43°32′55′′ W, 1220, m.a.s.l.). The worms were housed in thermoplastic polystyrene (PS) terraria (370 × 240 × 280 mm) with a window covered with a metallic net as air vent. The temperature varied between 18 and 23 °C. The terraria received artificial illumination with standard daylight fluorescent lamps and had a 3–4-cm layer of leaf litter extracted from the original habitat of the specimens. This litter contained small invertebrates that are the natural food of the worms. The humidity in terraria was kept close to saturation all the time.

### Optical microscopy

Tissues were fixed in 10% neutral-buffered formalin, processed with paraffin wax, sectioned at 6 μm and stained with haematoxylin and eosin for histological examination^42^,^43^. We used optical microscopy (Olympus BX41, Olympus camera E330-ADU1.2x, software Olympus Studio 2) to obtain the geometrical parameters involved in the reservoir and papilla duct, and to analyse the tissue structure at papilla and reservoir level.

### Electron microscopy

Tissue samples kept in 70% alcohol were collected for scanning electron microscopy (SEM). Selected samples were re-hydrated and cleaned with a 3% solution of sodium hypochlorite (NaClO) and distilled water. They were dehydrated with an increasing alcoholic series: 30, 50, 70, 80, 90 and 100%, respectively. Later they were critical point dried in a Baltec CPD 030 and then coated with gold (~20 nm thick) in a Denton Desk IV gold sputter system. Specimens were imaged in a SEM (Leica, LEO 440). The morphological terminology for structures follows the one in ref. 44^44^.

### Image analysis

The measurement of squirting times (Supplementary Table 1) was performed using high-speed imaging of *P. solorzanoi* during squirt. We elicited the attack from the specimen using a gentle paint brush on its back. We have used a Casio EX-ZR200 camera capable of capturing colour movies up to 1,000 f.p.s. For speed measurement (Fig. 1f and Supplementary Fig. 2) we have used two cameras; one at 30 f.p.s. (lateral view) and a second high-speed camera (Casio EX-ZR200) at 480 f.p.s. The first camera allowed us to measure the distance between the worm and the high-speed camera, and with this data and the pixel location from the high-sped camera we computed the Euclidean distance between the papilla and the jet tip. The time evolution of the slime jet tip is shown in Supplementary Videos 1 and 2. To measure oscillations in the synthetic system, we have used a Phantom Miro 310 high-speed camera. We used up to 8,000 f.p.s., as in this case we are free to use high-intensity illumination to capture detailed information about the oscillation amplitude and frequency (Fig. 3 and Supplementary Fig. 8).

### Micropipe fabrication and characterization

The fabrication of soft microtubes has been accomplished by standard fabrication techniques supplemented with some additional new techniques. The same method was used to fabricate rounded and rectangular cross-section channels. We began by fabricating a template of the channel of dimensions *w*~1 mm (width), *h*~1 mm (height) and *L*~30 mm (length). This was done using a standard micro-mill (*φ*=1.0 mm), on Plexiglas at extremely low feeding rates (5 mm per min) to avoid defects on the structure. A picture of the channels obtained is shown in Supplementary Fig. 5.

To make it easier to peel off the PDMS and the needle, we applied a soapy solution wash and let it dry; we repeated this procedure three times. The dried layer of soap^42^ does not allow PDMS to stick either to the template or to the needle. We have been able to produce micropipes of inner diameters (*d*) from 800 μm down to 300 μm in a reproducible way. We obtained these microchannels by using commercial needles of different sizes, which we mounted on a scotch tape that is covering the end of the channels, Supplementary Fig. 5b. We punctured this film with the help of a microscope (for proper centring) and later we introduced the required size needle through that centring hole. When pulling the needle out of the PDMS channel, the thin soap layer worked as a lubricant allowing easy slip without damaging the inner tube surface, Supplementary Fig. 5. Later we cleaned the tube using isopropyl alcohol.

We mixed Sylgard 184 in a ratio 25:1 to get a soft polymer for the pipes. We hand mixed for 3 min and then used a centrifuge for mixing and bubble removal. We repeated the previous steps three times. Once completing this step, we carefully filled the templates using a syringe and then degassed the sample for 40–60 min. We checked for bubbles and possible leaking in the tape wall and then placed it in an oven at 70° C for 15 h. The PDMS removal was manually done. Some samples showed small lateral films attached to the main pipe. These leftovers when existent were removed by using a scalpel under the microscope. We double checked the integrity of the final micropipes after isopropyl alcohol and nitrogen cleaning by visual inspection under the microscope.

### Model for fluid–papilla interactions

The motion of a pipe conveying fluid whose motions are restricted to be planar can be described by the following equation^29^:


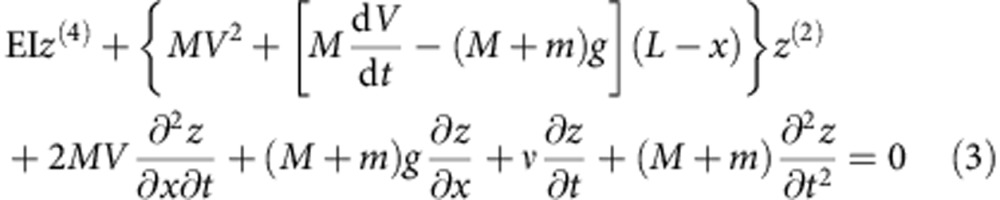


where *z*(*x*,*t*) is the lateral motion of the beam centre line, *M* the fluid mass per unit length, EI the flexural rigidity, *m* is the tube mass per unit length, *L* is the tube length, *V* is the fluid speed, *g* acceleration of gravity and *ν* is a phenomenological damping factor. We must emphasize that regardless of the cross-section shape the relevant quantity is the bending stiffness *B=*EI.

The boundary conditions in this problem are *z*(0,*t*)=0, *z*^(1)^(0,*t*)=0, *z*^(2)^(*L*,*t*)=0 and *z*^(3)^(*L*,*t*)=0. The relevant physical parameters needed to specify the parameter space for this problem can be found in equation (3) in a dimensionless form. This is easily achieved by using the dimensionless time and coordinates *η*=z/*L*, *χ*=*x*/*L*, *u*=*V*/*u*_0_ and *τ*=*t*/*τ*_0_. Where *τ*_0_ is the bending time scale, and *u*_0_ is a characteristic speed. The dimensionless equation reads:


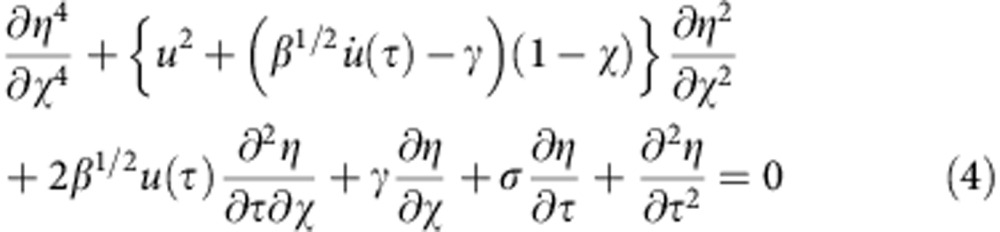


### Numerical simulation

We have solved equation (4) using different numerical approaches. We did compare different methods as the coefficients of the PDE are not constant and may abruptly change as a function of time. The results we show were obtained using the Garlekin method. We use as basis the set of functions provided by the problem:


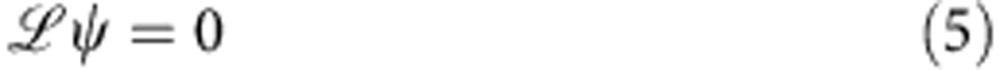


with the boundary conditions (BC) *ψ*(0)=0, *ψ*′(0)=0, *ψ*^(2)^(1)=0 and *ψ*^(3)^(1)=0. Where:


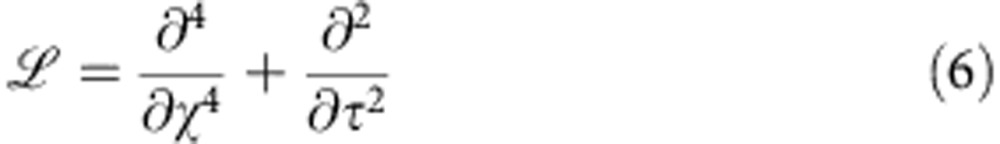


This problem endows us with a good expansion basis that fulfils the BC. After using the anzats,


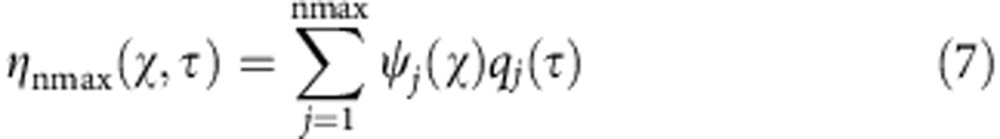


and using the inner product properties of 
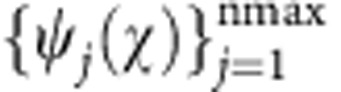
 over the domain [0,1], we reduced equation (4) into a linear system using the first *nmax* basis functions. We emphasize that equation (4) captures well the onset of the instability. However, the oscillation amplitude does not saturate without including nonlinear terms in curvature^29^. To keep the model simple, we augmented equation (4) by a confining term *αη*^2^, and included the experimentally found damping *σ* (Supplementary Fig. 6).

## Author contributions

A.C. brought together the collaboration, designed and built experiments, performed imaging and numerical simulations. P.M. and A.C. performed the microfluidic experiments and carried out the fluid-dynamics analysis. L.M. proposed the link between the biological problem and the artificial one, suggested the physical mechanism. B.M.-B., J.M.-N. and C.S.C. provided the velvet worms, performed and interpreted microscopy, and carried out the biological research. All authors contributed in writing the paper.

## Additional information

**How to cite this article**: Concha, A. *et al*. Oscillation of the velvet worm slime jet by passive hydrodynamic instability. *Nat. Commun*. 6:6292 doi: 10.1038/ncomms7292 (2015).

## Supplementary Material

Supplementary Figures, Supplementary Tables, Supplementary Notes and Supplementary References Supplementary Figures 1-9, Supplementary Tables 1-2, Supplementary Notes 1-8 and Supplementary References

Supplementary Movie 1 Movie showing the squirt of a red *Peripatus Solorzanoi* was recorded at 480 fps, and the largest shoot recorded by us was produced. In this case the maximum shooting distance was 0.54 m. We determined v_max_ ~ 5 m s^−1^ and the squirted volume ΔV ≈ 10μL.

Supplementary Movie 2 Movie showing the squirt of a White *Peripatus Solorzanoi* was recorded at 480 fps. This peripatus attacked the camera directly, showing its ability to direct the squirt using slow body and papilla motions, before squirting. In this case the camera was at a distance *d_camera_* = 25 cm away from the peripatus head.

Supplementary Movie 3 Movie showing the squirt of a White *Peripatus Solorzanoi* was recorded at 30 fps. This is the same attack shown in slow motion in Supplementary Movie 2, but from a different angle.

Supplementary Movie 4 Movie showing how the synthetic tube becomes unstable for a typical squirt dynamics and β = 0.25. The flow of water is increased until *u_max_* = 6.7 and then decreased.
