# Supplementary material for: Oscillation of the velvet worm slime jet by passive hydrodynamic instability
Source: Nat Commun. 2015 Mar 17;6:6292. doi: 10.1038/ncomms7292 (PMC4382676; doi:10.1038/ncomms7292)
Supplement: Supplementary Figures, Supplementary Tables, Supplementary Notes and Supplementary References — Supplementary Figures 1-9, Supplementary Tables 1-2, Supplementary Notes 1-8 and Supplementary References [file ncomms7292-s1.pdf]

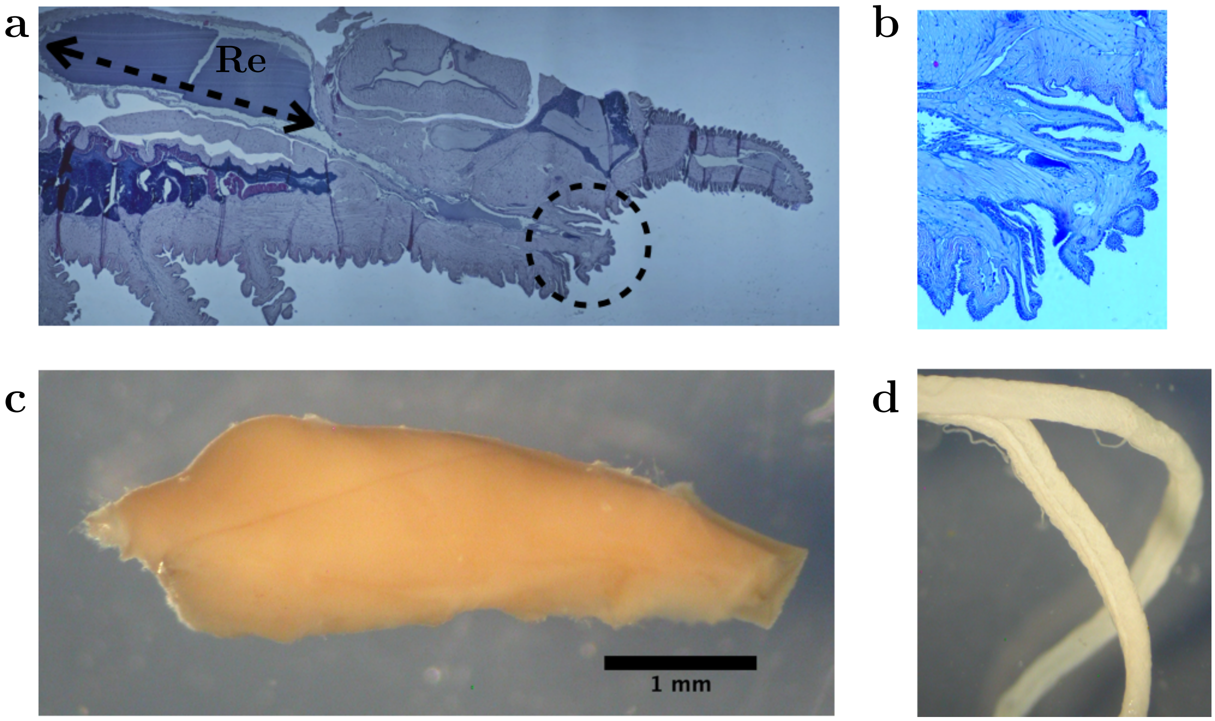

Supplementary Figure 1: **Optical Microscopy.** **a**, Longitudinal cut of a *Peripatus solorzanoi*. Dotted arrow mark the length $L = 13.0 \pm 0.2$ mm, of the widest part of an almost depleted reservoir, **Re**, (dark cylindrical area). Its diameter $D = 3.7 \pm 0.2$ mm. The reservoir becomes narrow and ends in the oral papilla (circled region). **b**, Zoom in of the oral papilla, circled region in Supplementary Fig.1**a**. We note that these dimensions are smaller than in the alive specimen as specimens contract when stored in formalin. Indeed, just after dissection the reservoir length was $L = 30 \pm 1$ mm. **c**, Solidified slime obtained from a different specimen. In this case $D = 1.9$ mm in the wider part. This 3D template clearly shows the difference between the narrow canal (leftmost region) and the center of the reservoir. **d**, Long reservoir showing muscular structure similar to the one reported by Baer *et al.*[1].

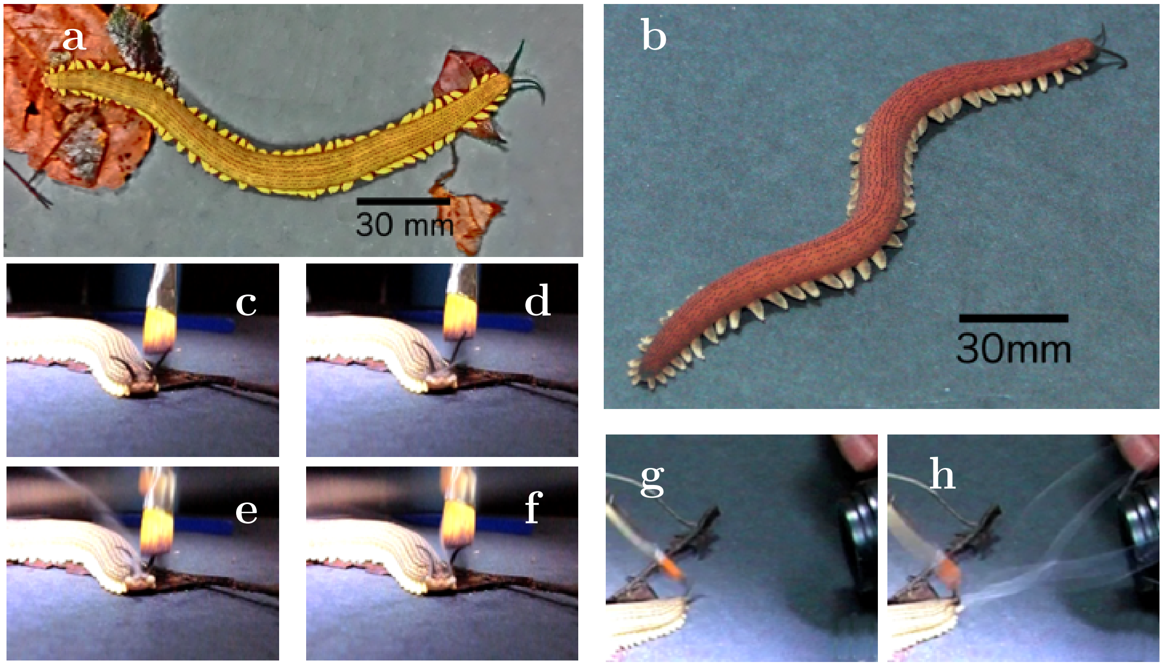

Supplementary Figure 2: **Worm attack and sizes.** **a**, *Peripatus solorzanoi* of sizes $\sim$ 18 cm, and **b** of size $\sim$ 17 cm are shown. **c**-**f**, Snapshots of a 480 fps (frames per second) video are shown (The attack was complete in $t_{squirt} \sim 60$ ms). To stimulate the attack we used a soft paintbrush, and light. **g**, **h**, side view of the attack taken with a 30 fps camera. Using information from both cameras we obtained data for Fig.1-f (Main text).

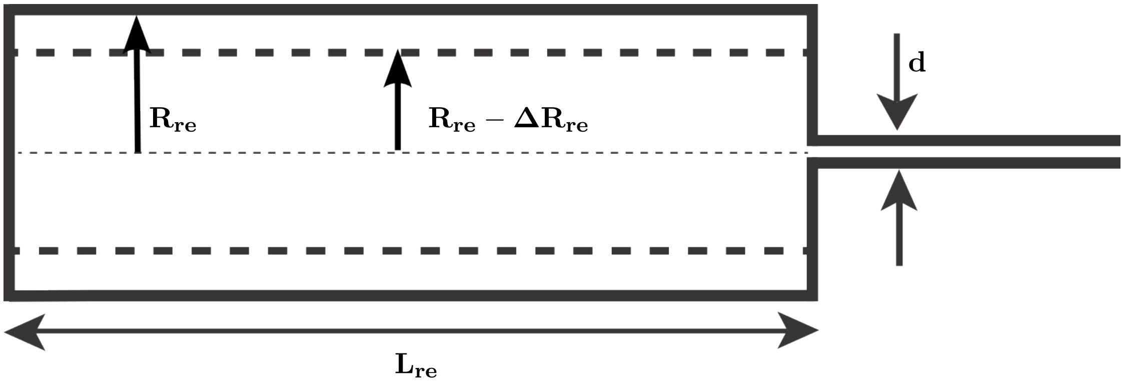

Supplementary Figure 3: **Syringe model diagram.** The big cylinder (solid line), of radius R_{re} represents the reservoir before contraction. After contraction the reservoir walls contract to a radius $R_{re} - \Delta R_{re}$. The small channel represents the canal through which the slime transits until reaching the oral papilla. The ability of the worm to squirt to very high speeds $V \sim 5$ m s^{-1} strongly depends on the ratio between reservoir and canal radius.

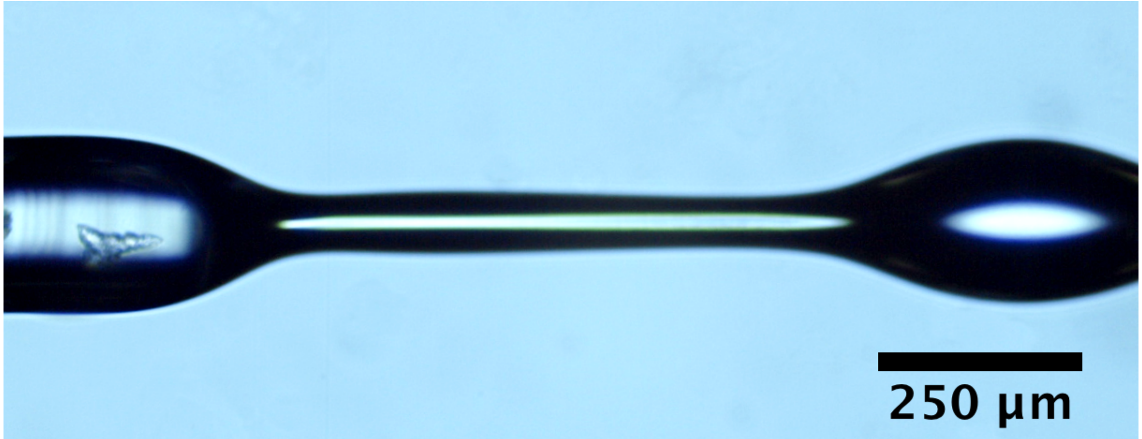

Supplementary Figure 4: **Fiber and beads.** Solidified jet (fiber) with typical structure shown across all specimens; a central fiber decorated by beads. These beads on a string effect is the consequence of the interplay between capillary, elastic, viscous, and inertial forces[2]. The beads on a string formation dynamics is cutoff by the drying process.

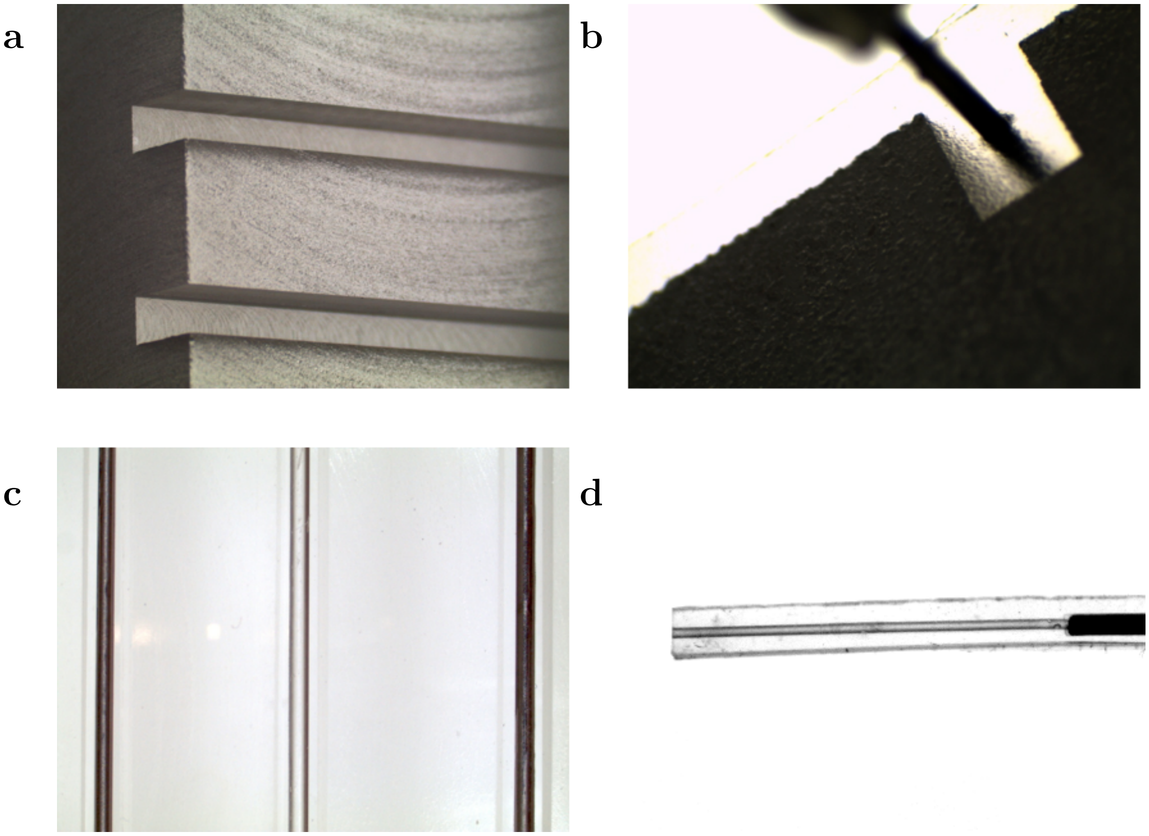

Supplementary Figure 5: **Template for micro-pipe production.** **a**, The acrylic template used to shape the micro-pipes. Channel width 1.6 mm, and depth 2 mm. **b**, Scotch tape centering technique used for holding the needles. Needle diameter 0.51 mm. **c**, Channels filled with PDMS and with and without needles. Needle diameter 0.81 mm. **d** After peeling off the channels and mounting the micro-pipe in a rigid rounded needle to form a cantilever. Needle diameter 0.51 mm, channel diameter 300 μm.

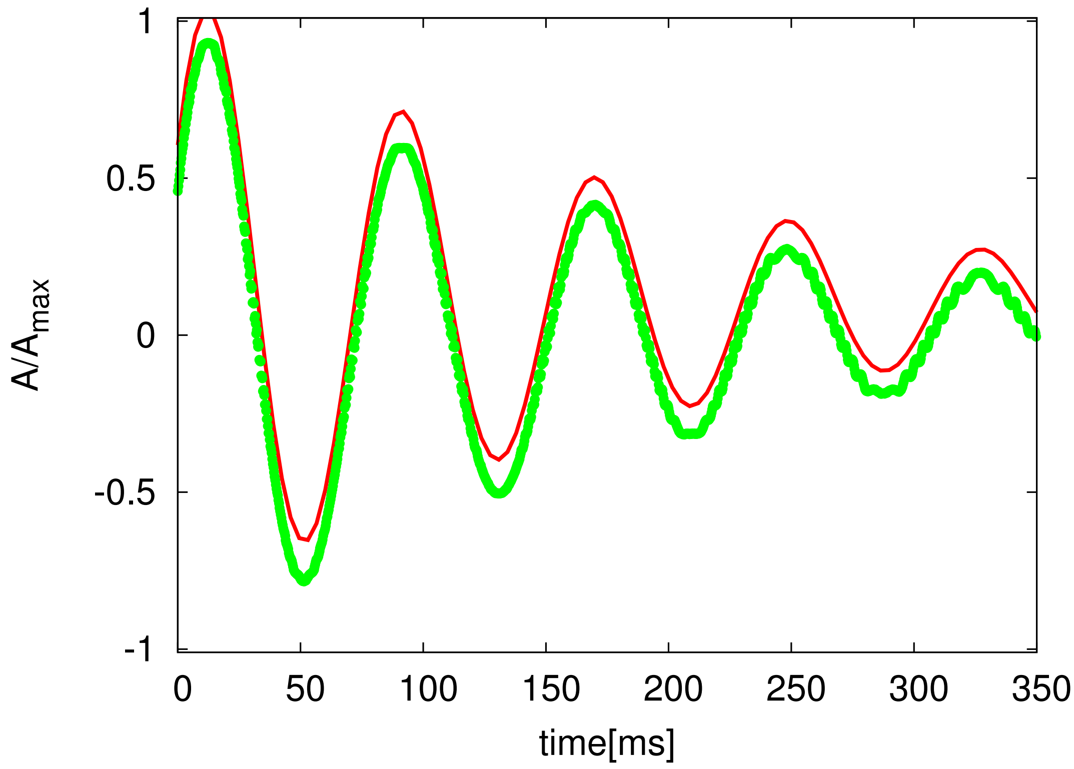

Supplementary Figure 6: **Free oscillations of the artificial papilla. a** After a slight perturbation, we recorded the damped oscillations of the cantilever. Imaging was done at 8000 fps. Experimental data (Green dots) were fitted to a damped dynamics $A = A_{max}e^{-\nu t}\cos(\Omega t + \delta)$ obtaining $\Omega = 79.78$ s^{-1}, $\delta = -1.04$, $\nu = 5.35$ s^{-1}. The red line is the best fit result were a small vertical offset was introduced for clarity.

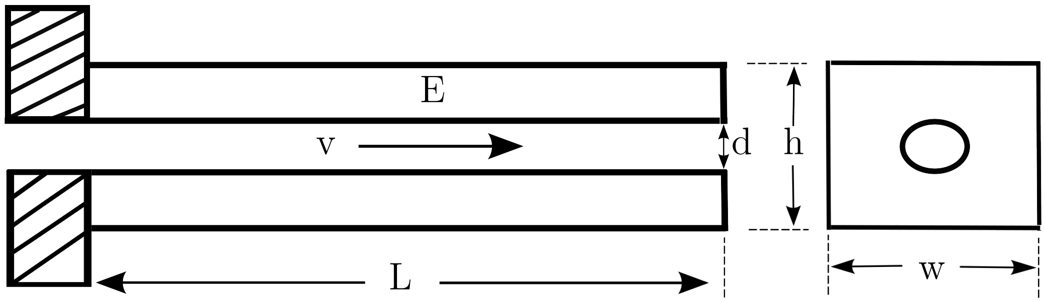

Supplementary Figure 7: **Synthetic papillae model.** A soft tube made out of Polydimethylsiloxane (PDMS) of Young's modulus, E. Its dimensions are d (inner diameter), h (tube height), w (tube width), L (papilla length), and V (liquid speed). In the experiment shown in Supplementary Movie 4, $L = 9.8$ mm, $w = 1.6$ mm, $h = 1.42$ mm, $u_0 = 1.4$ m s^{-1}, $\tau_0 = 0.014$ s.

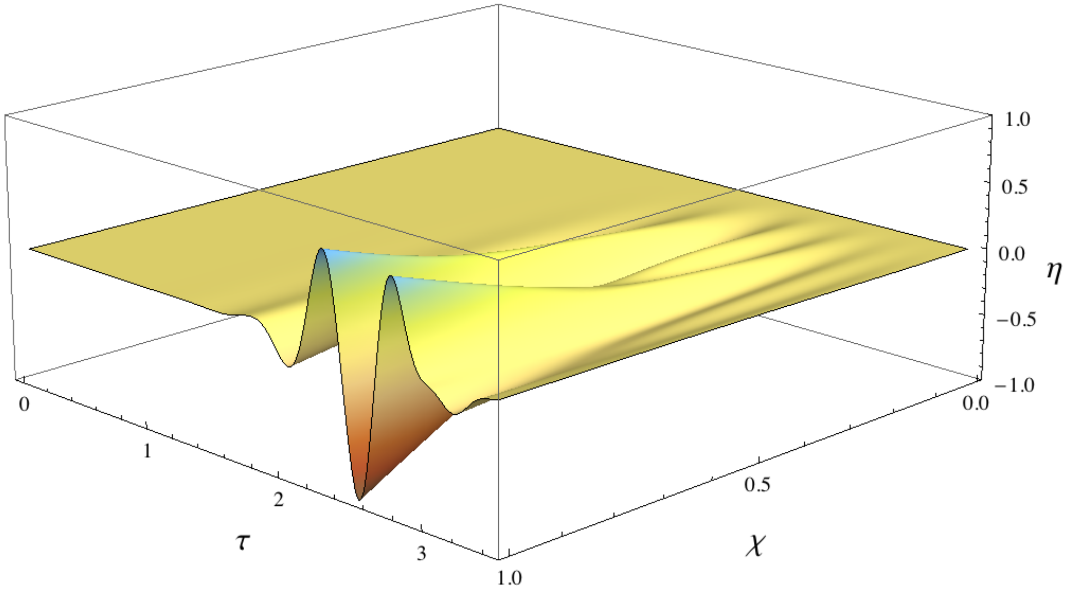

Supplementary Figure 8: **Spatio-temporal numerical evolution.** Numerical simulation result for artificial papilla motion. Results shown were obtained using Garlekin's method Eq. 3 (Main Text). Parameters used are $\beta = 0.24$, $u_{max} = 6.7$, $\tau_{max} = 3.0$. The χ-axis corresponds to the dimensionless coordinate along the papilla and the τ-axis corresponds to the dimensionless time of the system. The vertical axis corresponds to the dimensionless deformation amplitude η (See Methods).

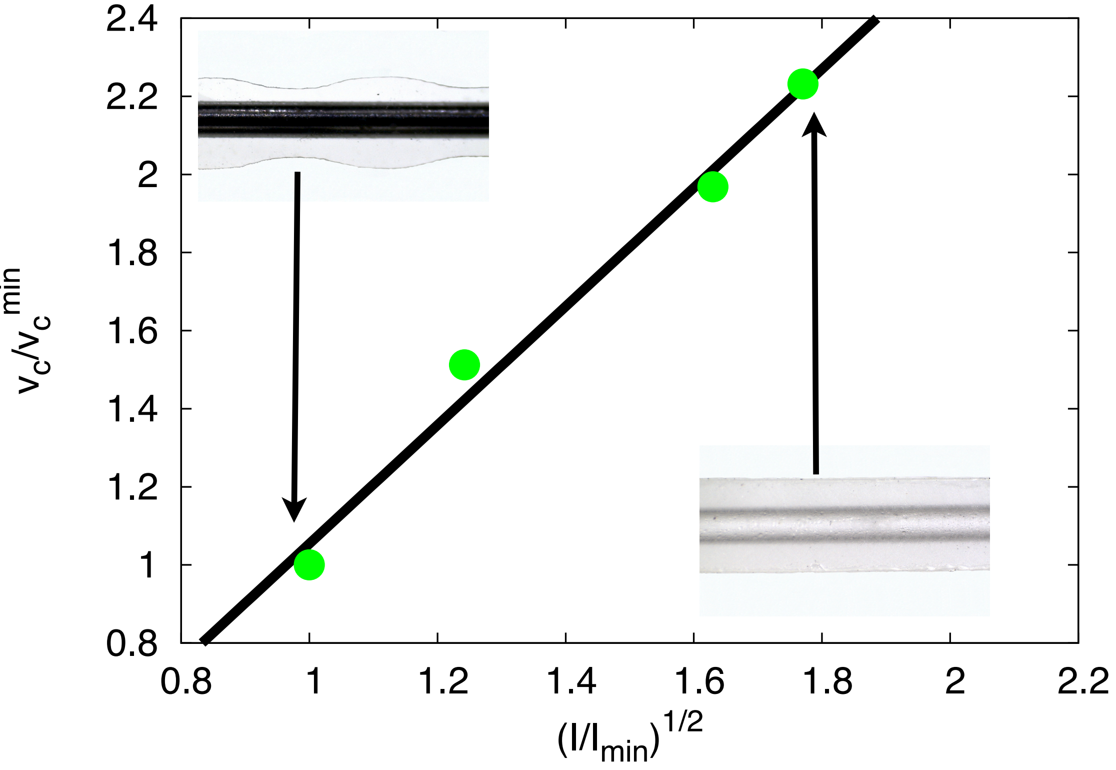

Supplementary Figure 9: **Critical velocity in accordion shaped micro tubes.** The critical velocity for pipe oscillations decreases when there are weak points. We show the rough micro tube before extracting the needle for clarity. The inner diameter is $d = 0.81$ mm. The horizontal axis corresponds to the square root of the ratio between the moment of inertia I, and the smallest local moment of the inertia for pipes used in this experiment I_{min}. The vertical axis is the ratio between the critical speed for a sample V_c, and the smallest critical speed V_c^{min} among all samples. The solid black line is the best linear fit consistent with our scaling arguments.

Specimen	fps	Active Phase	Δt $[s]$
Red 1	240	18	0.075
Red 1	240	13	0.054
Red2 (Left papilla)	480	30	0.063
Red2 (Right papilla)	480	37	0.077
White 1	480	25	0.052

Supplementary Table I: Squirt times obtained by using high speed video. Data from three giant specimens was obtained. Two red and one white. For specimen Red 2 we specify the papilla position as the liquid jet is not squirted at the same time in both papilla. The active phase column shows the number of fps where slime is actively being ejected from the papilla.

$\delta[10^{-4}\text{m}]$	$F[10^{-5}\text{N}]$	$E[\text{kPa}]$
7.5	17	41.7
10	22	40.5

Supplementary Table II: Force-deflection data used to compute Young's modulus (Each measurement δ was repeated three times). One papilla was used from the red *Peripatus solorzanoi*. Papilla deflection δ was measured as function of the magnetic force F. This allowed us to find[3] the Young's modulus E.

Supplementary Note 1: Optical Microscopy.

We have used optical microscopy to obtain the geometry of the reservoir and papilla system, as well as to analyze the tissue structure at papilla and reservoir level.

In Supplementary Fig.1 **a** − **b**, a longitudinal cut at middle reservoir level clearly shows that the specimen anatomy has a large reservoir (**Re**) that ends in a narrow canal (black arrow). That narrow canal leads to the opening of the oral papilla (See Fig. 1 main text). This geometry is in contrast to previously reported anatomical studies[4] where no mention to the sudden cross section change has been reported. The inner dimensions and 3-D structure of the reservoirs were directly confirmed as a solidified template of it was found during dissection, Supplementary Fig.1 **c**. The Onychophora slime was found in a solid state confirming the dimensions obtained by microscopy. The ratio between D and d is ~ 20. This provides a simple, syringe-like mechanism that permits high squirting velocities at oral papilla level.

Supplementary Note 2: Average squirt times.

In Supplementary Fig.2 examples of Onychophora and the attack process are shown. Analyzing videos of the squirting process for several specimens we counted the number of frames (fps: frames per second) at which the liquid jet was being expelled from the oral papilla. Our results are summarized in Table I. From this data we get an average of $\Delta t_{ave} = 0.064 \pm 0.005$ s. As described in Table II, and consistent with field observations we

have seen that right and left oral papillae may squirt at slightly different times, leaving the full body contraction hypothesis on weak grounds.

Supplementary Note 3: Slow muscular contraction for fast squirt.

As reported in Supplementary Note 1, the squirting mechanism is composed of a large reservoir that has strong muscle fibers in a configuration that makes the wall a contractile object[1]. This reservoir is connected to a narrow duct through which the slime is expelled. Based on this information the basic model of the squirting system is much alike a simple syringe (See Supplementary Fig.3). The slime is stored in a large cylinder (reservoir) of radius $\mathbf{R_{re}}$ and length $\mathbf{L_{re}}$ that radially contracts and pushes its content all the way through the narrow canal until it reaches the external opening located at the oral papilla (Supplementary Fig.1 **a-b** and Supplementary Fig. 3). As shown in Supplementary Fig. 3 (Geometry supported from our own observations and studied by Baer *et al.*) the whole mechanism is composed by the reservoir that contracts radially, and glands that produce the slime which transits to the reservoir. For the specimens used, the reservoir length $\mathbf{L_{re}} \sim 3 \cdot 10^{-2}$ m, and its radius has a typical value $\mathbf{R_{re}} \sim 2 \cdot 10^{-3}$ m. According to our measurements of the dry fiber and the organ opening (See Supplementary Figs. 1,4), the average fiber diameter is $d \sim 150$ μm. Using a high speed camera (240-480 fps) we have also measured the fiber propagation speed which is not constant but of order $V \sim 3 - 5$ m s^{-1}.

Using the above mentioned information we can ask how large the reservoir radial contraction should be in order to produce such a fast liquid squirting. This speed allows this slow moving worm to capture fast moving preys such as fireflies, and crickets between others. A cricket has a typical scape speed[5] of order 2 m s^{-1} .

We proceed to provide simple formulae that relate the vesicle geometry and jet dynamics considering an open geometry. The vesicle volume before and after contraction are $vol_i = \pi R_{re}^2 L_{re}$ and $vol_f = \pi \left(R_{re} - \Delta R_{re}\right)^2 L_{re}$, where ΔR_{re} is the change in the reservoir radius due to muscular contraction. Considering that $\Delta R_{re}/R_{re} \ll 1$ the volume of liquid expelled is $vol_i - vol_f \approx 2\pi R_{re} \Delta R_{re} L_{re}$ and that volume should be equal to the one of the squirted slime. In our experiments fiber length $\mathcal{C} \sim 60 \sim 10^{-2}$ m, and its mean radius $r \sim 75\mu$m. (Supplementary Fig. 4). Volume conservation forces that $2\pi R_{re} \Delta R_{re} L_{re} = \pi r^2 \mathcal{C} \sim 10\mu L$

from where the radial contraction can be computed as

$$\Delta R_{re} = \frac{r^2\mathcal{C}}{2R_{re}L_{re}} \tag{S1}$$

$$\Delta R_{re} = \frac{\left(75\cdot 10^{-6}\right)^2 60\cdot 10^{-2}}{2\cdot 2.0\cdot 10^{-3}\cdot 3\cdot 10^{-2}} \approx 30\cdot 10^{-6}\text{m} \tag{S2}$$

This is the radial contraction of the reservoir. This means that the circular muscles must contract $\frac{\Delta R_{re}}{R_{re}} \sim 0.03$ in order to produce the needed change in volume. This contraction is well within biological capability[6–8].

The previous estimate is a consequence of overall volume conservation. However, the liquid slime is at a good approximation incompressible. Therefore, flow conservation holds. Balancing injected flow from the vesicle and squirting flow from the papilla we obtain:

$$V_{out} = \frac{2R_{re}\Delta R_{re}L_{re}}{r^2\tau} \tag{S3}$$

using Eq. S1 we find that an estimate for the squirting time is given by:

$$\tau = \frac{\mathcal{C}}{V_{out}} \tag{S4}$$

where τ is the typical time scale in which the liquid is actively expelled. Given that V_{out} has been measured from high speed movies (Supplementary Movies 1- 2) together with a standard videocamera for triangulation (Supplementary Movie 3), and ΔR_{re} was obtained from volume conservation (Eq. S1) we can extract the typical time scale for the vesicle contraction that allows the fast squirt .

$$\tau = \frac{60\cdot 10^{-2}}{5} = 0.12\ \text{s} \tag{S5}$$

which is well within the reach of the muscles found in Onychophora, and consistent with experimental observations (Supplementary Movies 1 and 2). The radial contraction speed is quite slow $\Delta R_{re}/\tau \sim 3\cdot 10^{-4}$ m s^{-1}.

Supplementary Note 4: Elastic properties measurements.

We determine the bending stiffness of the artificial papilla $B = EI$, and from that determine the Young's modulus, E, of the PDMS produced. We used a dynamical way to

measure B. Free oscillation frequency, Ω, of a PDMS cantilever was measured recording at 8000 fps. Considering only the first eigenvalue λ_0 for the cantilever problem, we can find B using

$$\Omega = \left(\frac{\lambda_0}{L}\right)^2 \sqrt{\frac{B}{\mu}} \tag{S6}$$

where μ is the linear mass density, and L is the beam length. From the beam geometry we know that the moment of inertia is $I = wh^3/12$. We use this information and its relation with B to compute E of the PDMS sample. Experimental damped oscillations, and the best fit to $A(t) = A_{max}\cos(\Omega t + \delta)\exp(-\nu t)$ are shown in Supplementary Fig. 6 from where $E = 288$ kPa, and $\nu = 5.35$ s^{-1} is a damping factor.

In order to determine Papilla Young's Modulus (after dissection), we attached a small steel bearing ball (1mm in diameter) to the tip of an oral papilla, and used a magnet that was perpendicular to the sample to pull it. Thus, we deformed an oral papilla using magnetic forcing[3] in a free end cantilever configuration. In this case $F = 3\delta EIL^{-3}$, where F is the magnetic force, and $L = 3.0$ mm, the papilla length. We obtained $E \sim 40$ kPa (Supplementary Table II). This large value could be due to post-mortem rigidity combined with dry slime at the inner part of the tube.

Supplementary Note 5: Estimates of physical parameters.

The parameters that naturally emerge when obtaining the dimensionless Eq. 4 are: β, and γ corresponding to the ratio between the masses of liquid versus the total mass of the system, and the ratio between elastic forces and weight, respectively. Furthermore, typical speed u_0 and time τ_0 scales are needed to describe the dynamics of the system. We determine the values of these quantities to define the relevant parameter space in our experiments. The mass parameter for a tube of circular cross section is:

$$\beta = \left(\frac{M}{m+M}\right) = \frac{1}{1+\left(\frac{D}{d}\right)^2} \tag{S7}$$

where $d \in \{0.15, 0.5\}$ mm, and $D \in \{0.3, 1.0\}$ mm giving $\beta \sim 0.25$ for the Onychophora specimens used in our experiments.

The characteristic velocity parameter is defined by:

$$u_0 = \left(\frac{EI}{M}\right)^{1/2} \frac{1}{L} \tag{S8}$$

for $d = 0.5$ mm, $D = 1.0$ mm, and $E = 40$ kPa resulting into $u_0 \approx 0.51$ m s^{-1}. We have measured jet speeds $V \in [3.2, 5.0]$ m s^{-1}. Therefore, the dimensionless parameter $u \in [6.3, 9.8]$.

$$\tau_0 = \left(\frac{m+M}{EI}\right)^{1/2} L^2 \tag{S9}$$

for $d = 0.5$ mm, $L = 6.0$ mm, and $m + M = (\rho D^2/4)$ Kg m^{-1}. Thus, the elastic time scale is $\tau_0 \approx 0.023$ s. The typical squirt time is $\Delta t_{ave} = 0.064$ s, from where the dimensionless squirt time is $\tau_{max} = \Delta t_{ave}/\tau_0 \sim 3.0$.

The parameter characterizing the influence of gravity in this system is

$$\gamma = \frac{(M+m)g}{EI} L^3 \tag{S10}$$

In our experiments, $\gamma \in [0.2, 0.8]$, showing that the role of gravity is negligible compared with the centrifugal term $\sim u^2$.

For the case of synthetic papilla we have chosen a rectangular cross section in order make visualization easier. In this case

$$\beta = \left(\frac{M}{m+M}\right) = \frac{\pi D^2}{4wh} \tag{S11}$$

in the experiment shown in Fig.3 (main text), $V_{max} = 9.4$ m s^{-1}, $h = 1.4$ mm, $w = 1.6$ mm, and $E = 288$ kPa. Resulting in physical parameters $\beta = 0.24$, $u_0 = 1.40$ m s^{-1}, $\tau_0 = 0.014$ s, and $\gamma = 0.21$. Dimensionless squirt time is $\tau_{max} = t_{max}/\tau_0 \sim 3$, and $u_{max} = 9.4/1.40 = 6.7$ comparable to the one found for the real specimen.

Using these parameters obtained from our experimental data we obtained a spatio-temporal plot shown in Supplementary Fig.8.

To further characterize the natural and artificial systems we introduce an elasto-fluidic Reynolds number as:

$$Re^{EF} = \frac{\rho u_0 d}{\mu} \tag{S12}$$

where ρ, and μ are the liquid density and the dynamical viscosity respectively. The dynamical viscosity of the Onychophora slime is unknown up to date. However, it should not be far from the value for water as before to enter in contact with air it is composed of 90 percent of water, and the remnant 10 percent is made out of proteins, sugars, lipids and nonylphenol[9]. Thus, our estimates for the natural system is:

$$Re^{EF} = \frac{10^3 \text{kg m}^{-3} \cdot 0.51 \text{m s}^{-1} \cdot 0.5 \cdot 10^{-3} \text{m}}{8.9 \cdot 10^{-4} \text{Pa s}} \sim 290 \tag{S13}$$

and for the artificial one

$$Re^{EF} = \frac{10^3 \text{kg m}^{-3} \cdot 1.40 \text{m s}^{-1} \cdot 0.81 \cdot 10^{-3} \text{m}}{8.9 \cdot 10^{-4} \text{Pa s}} \sim 1250 \tag{S14}$$

Supplementary Note 6: The effect of an accordion like external structure.

In order to analyze the effect of local changes in the bending stiffness EI we built micro tubes with rectangular cross section, but with variable width, w (Supplementary Fig. 7). The thickness was constant $h = 2.2$ mm. The modulation was a sinusoidal pattern where the maximum always reached the same point. That is, the maximum width of all samples was constant ($w = 2.0$ mm), but the local width of the pipe wall varied as shown in Supplementary Fig. 9.

In order to find V_c for these samples we repeated the procedure shown and described in Fig. 3 (Main text). The only difference is that images were acquired at 3200 fps for better image resolution.

The control parameter in Supplementary Fig. 9 is the ratio $(I/I_{min})^{1/2}$, where I_{min} is the smallest local moment of the inertia for pipes used in this experiment (See top left image Supplementary Fig. 9). We used this dimensionless parameter as we know that the typical speed in this system is:

$$u_0 = \left(\frac{EI}{M}\right)^{1/2} \frac{1}{L} \tag{S15}$$

Therefore, when keeping M, E, and L constant, $V_c \sim I^{1/2}$. Therefore, $V_c/V_{min} \sim (I_c/I_{min})^{1/2}$. We have used as reference $V_{min} = 4.7$ m s^{-1} corresponding to the lowest critical speed measured in the micro pipe with the largest amplitude of the modulation (See leftmost inset Supplementary Fig. 9). Our data shows the onset of the instability occurs at lower fluid speeds when weak points are present in the micro pipes.

1. Baer, A. & Mayer, G. Comparative anatomy of slime glands in onychophora (velvet worms). *Journal of morphology* **273**, 1079–1088 (2012).
2. Bhat, P. P. *et al.* Formation of beads-on-a-string structures during break-up of viscoelastic filaments. *Nature Physics* **6**, 625–631 (2010).
3. Savin, T. *et al.* On the growth and form of the gut. *Nature* **476**, 57–62 (2011).
4. Manton, S. M. & Heatley, N. G. Studies on the onychophora. ii. the feeding, digestion, excretion, and food storage of peripatopsis with biochemical estimations and analyses. *Philosophical Transactions of the Royal Society of London. Series B, Biological Sciences* **227**, 411–464 (1937).
5. Read, V. & Hughes, R. Feeding behaviour and prey choice in macroperipatus torquatus (onychophora). *Proceedings of the Royal society of London. Series B. Biological sciences* **230**, 483–506 (1987).
6. Kaya, M. & Higuchi, H. Nonlinear elasticity and an 8-nm working stroke of single myosin molecules in myofilaments. *Science* **329**, 686–689 (2010).
7. Tashiro, N. Mechanical properties of the longitudinal and circular muscle in the earthworm. *Journal of Experimental Biology* **55**, 101–110 (1971).
8. Hoyle, G. & Del Castillo, J. Neuromuscular transmission in peripatus. *The Journal of Experimental Biology* **83**, 13–29 (1979).
9. Benkendorff, K., Beardmore, K., Gooley, A., Packer, N. & Tait, N. Characterisation of the slime gland secretion from the peripatus, *Euperipatoides kanangrensis* (onychophora: Peripatopsidae). *Comparative Biochemistry and Physiology Part B: Biochemistry and Molecular Biology* **124**, 457–465 (1999).
